# A Novel MSH6 Gene Variant in a Lynch Syndrome Patient with Lipomas

**DOI:** 10.3390/clinpract13020047

**Published:** 2023-04-07

**Authors:** Ana Paula Giannoni, Ina Sevic, Fernanda Parenti, Laura Alaniz

**Affiliations:** 1 Centro Médico de Cirugía (CmC) Famyl, Clínica Centro-Junín, Junín B6000, Argentina; 2 Laboratorio de Microambiente Tumoral, Centro de Investigaciones Básicas y Aplicadas (CIBA, UNNOBA), CIT NOBA, Universidad Nacional del Noroeste de la Provincia de Buenos Aires, Consejo Nacional de Investigaciones Científicas y Técnicas (UNNOBA-CONICET), Junín B6000, Argentina

**Keywords:** MSH6, Lynch syndrome, lipomas, genetic screening, cancer risk

## Abstract

Colorectal cancer is one of the most frequently occurring cancers today, with a large percentage of cases having a hereditary basis. Lynch syndrome is the most common cause of hereditary colorectal cancer. The genetic defect characteristics of this syndrome involve mutations in mismatch repair (MMR) genes, which result in microsatellite instability. Early detection of the mutation can help evaluate the cancer risk and, consequently, a proper course of clinical management for the person harboring the mutation. Herein, we describe the first report of a c.1458dup (p.Glu487*) new mutation in a 53-year-old colorectal cancer patient with diagnosed Lynch syndrome. Additionally, the existence of lipomas in this patient and his family could be related to this syndrome. Further investigation may provide a possible visual clue that can indicate a need for genetic screening.

## 1. Introduction

Colorectal cancer is one of the most commonly diagnosed cancers in the world, with nearly 2 million new cases (10% of all cancer sites) and nearly 1 million deaths (9.4% of all cancer sites) globally in 2020 [1]. The most common cause of hereditary colorectal cancer is Lynch syndrome, which accounts for approximately 3–5% of all colorectal cancer cases [2,3]. Lynch syndrome is an autosomal dominant hereditary syndrome, and it is proposed that cancer development in Lynch Syndrome patients occurs due to the pathogenic germline mutation in one of the DNA mismatch repair (MMR) genes (MLH1, MSH2, MSH6, or PMS2), or to EPCAM 3` gene deletion. These mutations lead to malfunctions of the MMR system, which consequently fails to correct mismatched bases and increases the chance of mutations. Additionally, a deficient MMR system can often lead to microsatellite instability. All of these factors increase the probability of developing cancer driver mutations, and, therefore, increase the chance of the development of cancer. For this reason, carriers of one of the pathogenic mutations of MMR genes are considered to have an increased risk of colorectal and certain other cancers, such as cancer of the endometrium, ovaries, prostate, stomach, pancreas, etc., over their lifetimes [2,3,4].

Early detection of these mutations and a diagnosis of Lynch syndrome are crucial, as they provide a way for the mutation carriers to implement the screening guidelines recommended for cancer risk management, such as colonoscopy, which allows the patient to start treatments in time and thus lower the colorectal cancer mortality rate. Herein, we report a new mutation that, to our knowledge, has not been previously described in colorectal cancer patients and which can help with early detection of cancer risk.

## 2. Case Presentation

**Figure 1:**
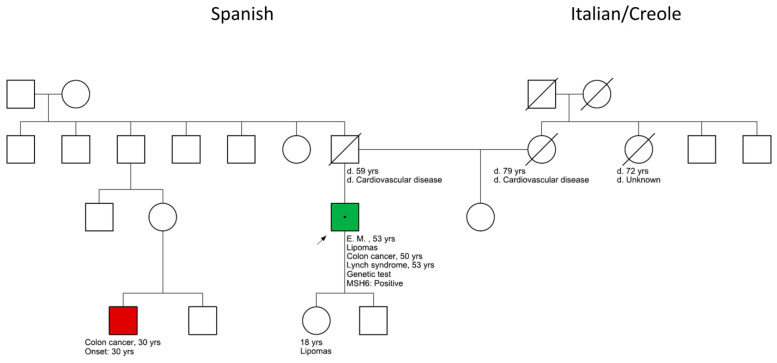
Family pedigree with known medical history. Ages represent (i) age of death for deceased subjects or (ii) age at the time of medical history collection for living family members.

A 53-year-old patient presented a lipoma on the abdominal wall upon physical examination, and reported a history of two resections of lipomas in previous years on the back and left axillary lines. The colonoscopy control, at 40 years of age, showed a normal result, while at the age of 50, the patient was diagnosed with sigmoid colorectal cancer, requiring a left hemicolectomy. The patient has an 18-year-old daughter who presented lipomas with benign characteristics upon physical examination and a paternal third-degree relative who was diagnosed with colorectal cancer at the age of 30. The cousin additionally presents rosacea, but information about lipomas is unavailable from him (Figure 1). The patient did not receive any treatment before the surgery. With the exception of lipomas, the patient did not have any other comorbidities. The patient signed written consent, which was approved by the ethics committee (COENOBA Exp-1291/2021).

### 2.1. Tumor Biopsy

A tumor biopsy was performed, and the histological result of the pathological anatomy revealed a sigmoid poorly differentiated mucoid adenocarcinoma of a high histological grade (WHO). The microscopic tumor extension was serous inclusive, without vascular or lymphatic invasion. Circumferential margins were compromised, while proximal and distal margins were not. Pathological TNM staging classified the tumor as pT4. Twelve lymph nodes were examined and were found to be free of metastases (pT4 pN0). No suggestion of a possible distal metastasis lesion was found at this time. In addition, findings suggesting microsatellite instability (histological type) were discovered.

### 2.2. PREMM5 Risk Prediction

The PREMM5 model is a clinical prediction algorithm that evaluates the probability that one carries a germline mutation in the MLH1, MSH2, MSH6, PMS2, or EPCAM genes. This model also requires an individual to have a family history of colorectal or associated cancer. An algorithm was used with the PREMM5 risk prediction model (https://premm.dfci.harvard.edu/ accessed on 22 June 2022), which yielded a cumulative risk of 12.1% (greater than 2.5% is considered a high probability) [5,6].

### 2.3. Histological Results

**Figure 2:**
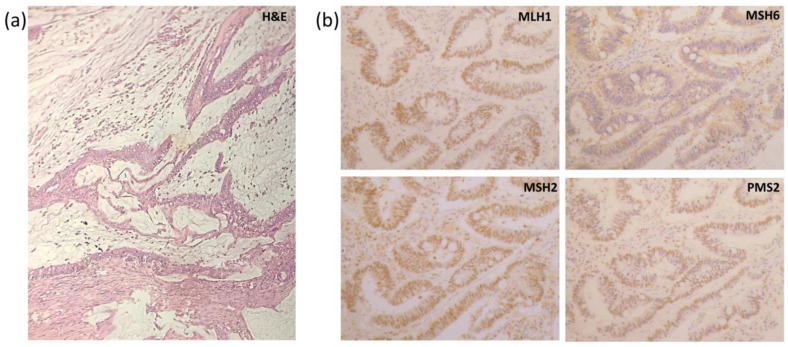
Histopathological analysis. (**a**) H&E staining; (**b**) immunohistochemical staining. Source: Unidad de Patología, Hospital Bonorino Udaondo, CABA, Argentina.

Considering that the patient had colon cancer at the age of 50 and that the PREMM5 risk prediction model showed high cumulative risk, immunohistochemistry (IHC) was performed to search for a deficit in the expression of DNA repair proteins (MLH1, MSH2, MSH6, and PMS2). The results confirmed a deficit of MSH6 (Figure 2):

Anti-hMLH1: intact nuclear positivity in tumor cells.

Anti-hMSH2: intact nuclear positivity in tumor cells.

Anti-hMSH6: loss of nuclear positivity in tumor cells.

Anti-PMS2: intact nuclear positivity in tumor cells.

Suspecting the possibility of Lynch syndrome, the patient was referred for genetic counseling.

### 2.4. Genetic Panel Results

The results up to this point showed that the patient presented a sigmoid poorly differentiated mucoid adenocarcinoma at an early age (≤50 years of age) and had a third-degree relative (paternal cousin) who developed colon cancer at 30 years of age. For this reason, the patient met the revised Bethesda criteria, but the lack of the third instance of cancer in his close family makes the Amsterdam criteria non-applicable. The Amsterdam criteria exclude most suspect cases of possible hereditary colorectal cancer, while the revised Bethesda criteria broaden the disease spectrum. Furthermore, the histological and molecular characteristics of the cancer tissue prompt a high level of suspicion of a possible hereditary non-polyposis colorectal cancer syndrome (possibly Lynch syndrome). Finally, IHC demonstrated the deficiency of MSH6 expression.

Due to all of the above, a germinal study of the following genes was performed to rule out or confirm the hereditary oncological pathology (possible Lynch syndrome): MLH1, MSH2, MSH6, PMS2, MUTYH, EPCAM, POLE, and POLD1.

A genetic panel was performed on the peripheral blood sample. The result showed a new pathogenic variant, a heterozygous mutation in the MSH6 gene c.1458dup (p.Glu487*), which has not previously been reported in LS patients. This variant inserts 1 nucleotide into exon 4 of the MSH6 gene, creating a frameshift and premature translation stop signal.

The following genes were also analyzed as a part of the genetic panel, and no pathogenic or likely pathogenic variants associated with an increased risk of hereditary colorectal, male breast, melanoma, pancreatic, prostate, or stomach cancers were identified: APC, ATM, BAP1, BARD1, BMPR1A, BRCA1, BRCA2, BRIP1, CDH1, CDK4, CDKN2A (p14ARF), CDKN2A (p16INK4a), CHEK2, EPCAM, GREM1, MITF, MLH1, MSH2, MUTYH, NBN, PALB2, PMS2, POLD1, POLE, PTEN, RAD51C, RAD51D, SMAD4, STK11, and TP53.

## 3. Discussion

Lynch syndrome patients present a genetic predisposition to and a higher probability of early-onset colorectal cancer. These patients also have an increased risk of other types of cancer, such as cancer of the endometrium, ovaries, prostate, stomach, pancreas, etc. Genetic testing for the diagnosis of this syndrome is critical because it not only allows for a more precise risk evaluation for cancer development, but also helps with clinical management, timing, and extent of surgery for patients with colorectal cancer [7,8].

Loss of the function of one of the MMR genes, MSH6, is a known mechanism of this disease. The MMR system repairs single base mismatches as well as small insertions and deletions, which occur mainly during replication [9,10]. Herein, we report a new mutation in this gene found in a colorectal cancer patient with a history of lipomas. This new mutation inserts 1 nucleotide in exon 4 of the MSH6 gene and, as a consequence, a frameshift is created, resulting in a premature translation stop signal. As a result, it is expected that this variant will result in a truncated protein with a loss of function or of the protein product. To our knowledge, this variant has not been reported previously in hereditary colorectal cancer patients. Additionally, the Genome Aggregation Database has not identified this mutation in the general population (gnomAD; 2022). Available from: https://gnomad.broadinstitute.org/ (accessed on 12 August 2022).

On the other hand, the patient presented a past medical history of lipomas upon physical examination. A Lynch syndrome variant, Muir–Torre (MTS), is characterized by the presence of sebaceous neoplasms and/or keratoacantomas, though the loss of MSH2 and MSH6 expression has also been reported [11]. A case report by Burris et al. reported a man with skin tags, a familiar history of different types of cancer, and an IHC with no functional MSH2 and MSH6, confirming the presence of MTS. In that article, the authors highlight the importance of taking into account detailed medical history of the patient and describe that their patient had a history of remotion of fatty tumors when he was a child [12]. For this reason, we considered lipomas to be an important part of our patient´s medical history, and suspect that the manifestation of lipomas could be associated with MTS.

Navarro et al. found this mutation in urethral cancer, while Flaum et al. also mention this mutation in endometrial cancer [13,14]. Taking into account these two reports and our colorectal cancer patient, the importance of this mutation in LS patients is clear. Considering the report by Burris et al. as well, in which they observed lipomas in their patient´s history, we considered that it to be important to report all possible physical indicators which could help in patient management. Our intention was to document this case with all the relevant details, with the hope that this report could help with patient management in some future case, mainly by taking into consideration this mutation and not disregarding the presence of lipomas.

In conclusion, to the best of our knowledge, this is the first report of a c.1458dup (p.Glu487*) mutation in a colorectal cancer patient. It could be introduced as a new variant for the Lynch syndrome screening, and, thus, help with early detection of cancer risk. On the other hand, the existence of lipomas in this patient and his family could be further investigated, because further findings may provide a possible visual hint indicating a need for genetic screening.

## Data Availability

The data supporting the findings in this study are available within the article.
